# NUDT15 R139C Variants Increase the Risk of Azathioprine-Induced Leukopenia in Chinese Autoimmune Patients

**DOI:** 10.3389/fphar.2018.00460

**Published:** 2018-05-07

**Authors:** Xiang Fei, Qing Shu, Huaijun Zhu, Bingzhu Hua, Shiying Wang, Ling Guo, Yun Fang, Weihong Ge

**Affiliations:** ^1^Department of Pharmacy, Nanjing Drum Tower Hospital, Nanjing, China; ^2^School of Basic Medicine and Clinical Pharmacy, China Pharmaceutical University, Nanjing, China; ^3^Department of Rheumatology and Immunology, Nanjing Drum Tower Hospital, Nanjing, China; ^4^Nanjing Drum Tower Hospital, Clinical College of Traditional Chinese and Western Medicine, Nanjing University of Chinese Medicine, Nanjing, China

**Keywords:** azathioprine, *NUDT15 R139C*, leukopenia, Chinese, autoimmune patients

## Abstract

The aim of this study was to investigate the influence of *NUDT15 R139C*, thiopurine S-methyltransferase (*TPMT*), and 6-TGN on azathioprine (AZA) induced leukopenia in Chinese autoimmune patients. Among 87 enrolled patients, 23 (26.4%) had leukopenia. The *NUDT15 R139C* variant was associated with leukopenia (*p* = 1.86 × 10^−7^; OR: 7.59; 95% CI: 3.16–18.21). However, *TPMT* genotype was not shown to be correlated with the incidence of leukopenia (*p* = 0.95). There was no significant difference of 6-TGN concentration between patients with or without leukopenia (*p* = 0.15) and no association was found in patients with *NUDT15 R139C* variants alleles (*p* = 0.62). Finally, we found that the range of 6-TGN concentrations in autoimmune diseases was much lower than the established 6-TGN monitoring range for inflammatory bowel diseases. Therefore, the variant of *NUDT15 R139C* is strongly associated with AZA-induced leukopenia in Chinese patients with various autoimmune diseases such as systemic lupus erythematosus, Sjögren's syndrome, etc.

## Introduction

Azathioprine (AZA) is a thiopurine prodrug commonly used as an immunosuppressive agent in the treatment of inflammatory bowel disease (IBD), systemic lupus erythematosus (SLE), Sjögren's syndrome (SS), and other autoimmune diseases (Bertsias et al., 2008; Ramos-Casals et al., 2010; Timmer et al., 2012, 2016; Janssens et al., 2013; Okon and Werth, 2013). AZA is relatively safe for clinical use, though several studies have reported up to 50% of patients discontinued AZA during long-term therapy, mainly due to the adverse drug reactions (ADRs) (de Jong et al., 2003; Jharap et al., 2010). Myelosuppression, one of the AZA-induced life-threatening adverse events, occurred in 3–17% of these patients (Boonsrirat et al., 2008; Ngo et al., 2011).

As a prodrug, AZA is nonenzymatically broken down to 6-mercaptopurine (6-MP), and then 6-MP is converted to 6-thioguanine nucleotides (6-TGN) or various other metabolites by a number of enzymes (Teml et al., 2007; Moon and Loftus, 2016). The predominant active metabolites 6-TGN cause cytotoxicity by interfering with *de novo* purine biosynthesis and modification of DNA structure after their incorporation into nucleic acids (Somerville et al., 2003). Finally, part of 6-TGN is hydrolysed to inactive metabolites by nucleoside diphosphate-linked moiety X-type motif 15 (NUDT15), which is one member of the nudix hydrolase enzyme family (Carter et al., 2015).

In this process, thiopurine S-methyltransferase (TPMT) metabolizes AZA to inactive molecules, and variant *TPMT* alleles (slow metabolizer) are associated with AZA-induced leukopenia with an elevation of 6-TGN level (Hiratsuka et al., 2000). The association between AZA-induced leukopenia and *TPMT* mutations is well-established. *TPMT* gene testing before AZA exposure is recommended by the US Food and Drug Administration (FDA) to predict adverse events and guide selecting doses of AZA (Relling et al., 2013). However, increasing studies found that the frequency of *TPMT* mutation is considerably lower in Chinese than in Caucasians, with the lowest frequencies observed in Chinese (about 0.9%; Fangbin et al., 2012; Zhu and Cao, 2012) compared with a higher incidence of AZA-induced leukopenia (27–41.3%; Connell et al., 1993). A recent study found that *NUDT15 R139C* was strongly associated with AZA-induced leukopenia in Koreans (Yang et al., 2014). Additionally, (Zhu et al., 2016) confirmed the association of *NUDT15 R139C* with early leukopenia in Chinese IBD patients commencing AZA treatment. These findings suggested that *NUDT15 R139C* is a potential genetic factor that is responsible for AZA-induced leukopenia in East Asian populations. Azathioprine is more widely used in various autoimmune diseases like SLE and SS compared to IBD. However, the association of *NUDT15 R139C* variants with AZA-induced leukopenia had not been reported in Chinese autoimmune diseases.

Moreover, the clinical efficacy of AZA had been reported to be correlated with erythrocyte levels of 6-TGN (Cuffari et al., 1996; Osterman et al., 2006). An improved clinical response to AZA has been reported in IBD patients when the concentration of 6-TGN was 235 pmol/8 × 10^8^RBC or higher (Dubinsky et al., 2000; Teml et al., 2007; Hanai et al., 2010). However, leukopenia occurred when higher 6-TGN levels(450 pmol/8 × 10^8^RBC) were achieved (Dubinsky et al., 2000). Another study in SLE patients found that clinical response can occur at lower 6-TGN levels than the target range established for IBD (Osterman et al., 2006). The distribution of 6-TGN levels in Chinese autoimmune diseases is still unknown.

Our study was aimed to investigate NUDT15 R139C, TPMT^*^3C, 6-TGN levels and explore their influence on AZA-induced leukopenia in Chinese autoimmune diseases (except IBD), retrospectively.

## Materials and methods

### Patient recruitment

A total of 87 patients with autoimmune diseases with AZA therapy for more than 2 months were recruited in the Drum Tower Hospital affiliated Nanjing University Medical School from Sep 1st 2016 to Apr 1st 2018. These patients exhibited various autoimmune diseases including SLE, SS, dermatomyositis (DM), and others. All subjects were Chinese, and their clinical characteristics are shown in Table 1. Exclusion criteria included patients who were concomitantly treated with blood transfusion or other immunosuppressants that could result in leukopenia such as cyclosporine or tacrolimus (FK506), or treatments potentially interfering with AZA metabolism including allopurinol, 5-aminosalicylates and diuretics. Moreover, insufficient function of heart, liver, or kidney and suspected infection were excluded. The clinical data of these patients were analyzed from medical records, mainly including age, sex, body weight, combination of drugs, erythrocyte sedimentation rate (ESR), white blood count (WBC), red blood count (RBC), neutrophil count (NEU), platelet (PLT), alanine aminotransferase (ALT), and aspartate transaminase (AST), with a specific focus on leukopenia. This study was conducted in accordance with the ethical guidelines and approved by the research ethics committee of Drum Tower Hospital affiliated Nanjing University Medical School, Nanjing, China (2017-101-01). All methods were carried out in accordance with the approved guidelines.

**Table 1:** Baseline characteristics of subjects in this study.

**Characteristics**	**Patient**		***p***
	**Leukopenia**	**Controls**	
No. of subjects (%)	23(26.4%)	64(73.6%)	–
Female (%)	23(100%)	56(87.5%)	0.001
Age (years)	37.0 ± 9.94	35.4 ± 10.25	0.636
AZA dose (mg/day)	58.3 ± 19.17	55.8 ± 16.13	0.285
WBC_0 w	5.5 ± 1.02	5.9 ± 1.12	0.197
NEU_0 w	3.9 ± 1.34	4.3 ± 1.57	0.068
**DISEASE**			
SLE	13	30	
SS	6	15	
Vasculitis	2	5	
Scleroderma	2	3	
DM	0	5	
CTD	0	3	
IgG4-RD	0	2	
AIH	0	1	
**OTHER ADVERSE EVENTS**			
Neutropenia	10(43.5%)	0(0%)	< 0.001
Severe hair loss	2(8.7%)	0(0%)	< 0.001
Vomiting	0(0%)	1(1.6%)	0.236

*Controls, the patients without leukopenia; No., number of; AZA, azathioprine; WBC_0w, the white blood cell counts at the initial of azathioprine treatment; NEU_0w, neutrophil counts at the initional of azathioprine treatment; SLE, systemic lupus erythematosus; SS, Sjögren's syndrome; DM, dermatomyositis; CTD, connective tissue disease; IgG4-RD, IgG4 related diseases; AIH, autoimmune hepatitis*.

### AZA treatment and adverse events

An initial dose of AZA at 50 mg per day was commonly prescribed and adverse events were checked at 7–14 days from the beginning of medication. Routine blood counts were required once a week in the first month, then twice a month until 12 weeks. Two milliliters of venous blood samples (EDTA anticoagulation) were obtained at least 8 weeks after administration of AZA or at the time when adverse events occurred for erythrocyte 6-TGN concentration measurement and gene testing. If patients developed any adverse drug events, the suspicious medication were stopped immediately and subsequent treatments were started by the responsible physician on a case-by-case basis.

AZA-induced leukopenia was defined as WBC of <3.5 × 10^9^/L. Leukopenia before 8 weeks, and after 8 weeks were defined as early and late leukopenia, respectively. Neutropenia was defined as NEU of <1.8 × 10^9^/L. Severe hair loss was defined as objective hair loss that patients may need to wear wigs or needed a few months to recover, and hepatotoxicity was regarded as ALT or AST >2-fold the upper normal limit without other causes. Gastrointestinal discomfort including nausea and vomiting.

The rheumatology specialists would confirmed the adverse events caused by AZA after carefully consideration. In order to rule out other causes, diagnostic criteria of leukopenia induced by AZA are as follows. Firstly, the initial WBC of enrolled patients is normal (WBC > 3.5 × 10^9^/L). Secondly, the time point of leukopenia happened is consistent with the acute phase of leukopenia caused by AZA (12 weeks). Thirdly, after withdraw of the AZA, the WBC of these patients who developed leukopenia would improve.

### Gene analysis

Total genomic DNA was isolated from peripheral leucocytes by a DNA extraction kit purchased from Promega (Madison, WI, USA). Genotyping for *NUDT15 R139C* (rs116855232) and TPMT^*^3C (rs1142345) were performed using Custom TaqMan®SNP genotyping assays (ID: C_154823200_10 and C___19567_20; Life Technologies, Carlsbad, CA, USA) in accordance with manufacture's information.

PCR was performed according to the manufacturer's instructions provided by Thermo Fisher Scientific. The PCR thermal cycling was as follows: initial denaturing at 92°C for 10s followed by 50 cycles of 15s at 92°C and 90s at 60°C for annealing and extension. Thermal cycling was performed using a LightCycler 480 system (Roche Diagnostics, Switzerland). Each 96-well-plate contained 87 samples of an unknown genotype and 2 reaction mixtures containing the reagents, but no DNA (quality control). The no template controls were necessary for the Sequence Detection System (SDS) signal processing, as outlined in the TaqMan Allelic Discrimination Guide. The genotypes were determined visually based on the dye component fluorescent emission data depicted in the X-Y scatter-plot of the SDS software.

### Blood concentration measurement of 6-TGN

The 6-TGN concentrations in erythrocyte were measured by high performance liquid chromatography, as previously described (Dervieux and Boulieu, 1998).

### Hardy–Weinberg equilibrium (HWE)

HWE analysis was performed on the research subjects by comparing the detected distribution of allele frequencies with the theoretical distribution estimated on the basis of the SNP allelic frequencies. *p* > 0.05 (Chi-squared statistics) was considered to indicate equilibrium.

### Statistic analysis

Statistic analysis and calculations were performed by SPSS 20.0 (SPSS Inc., Chicago, IL, USA) and Prism 6 (Graph Pad Software, La Jolla, CA, USA). Data for continuous variables were expressed as the mean ± SD, and those for categorized variables were expressed as frequencies. A one-sample Kolmogorov-Smirnov test was used to evaluate the normal distribution of 6-TGN metabolite concentrations. Categorical variables were compared using method χ^2^ or Fisher's test. A non-parametric Kruskal-Wallis H-test was used to evaluate the difference among independent groups. The sensitivity and specificity of *NUDT15 R139C* for detecting leukopenia and early leukopenia were calculated using the receiver operating characteristic curve (ROC). SHEsis (http://analysis.bio-x.cn/myAnalysis.php) was used to determine the deviation from the Hardy-Weinberg equilibrium. *p* < 0.05 was regarded as statistically significant.

## Results

### Patient characteristics

A total of 98 consecutive patients were enrolled in this study. Among them, 11 patients were excluded from this study by the following reasons: refusal to participate in this study (*n* = 5); taking immunosuppressant concomitantly with AZA (*n* = 3); incomplete data for AZA treatment (*n* = 3). A total of 87 patients (79 females and 8 males) were analyzed in the present study. The percentage of females was much higher than males because females are more susceptible to autoimmune diseases like SLE, SS. Patients exhibated various autoimmune diseases as follows, SLE (*n* = 43), SS (*n* = 21), vasculitis (*n* = 7), scleroderma (*n* = 5), DM (*n* = 5), connective tissue disease (CTD) (*n* = 3), IgG4 related diseases (*n* = 2), and autoimmune hepatitis (AIH) (*n* = 1). The baseline characteristics of these patients are summarized in Table 1. Leukopenia was observed in 23 patients (26.4%), 2 of them also experienced severe hair loss and 10 of them developed neutropenia during the follow-up visit.

The percentage of females who had severe hair loss and neutropenia was significantly higher than males (*p* = 0.001, *p* < 0.001, respectively). No significant differences were observed in the age, AZA doses, initial WBC and NEU counts between individuals with or without leukopenia (controls) (*p* > 0.05; Table 1).

*NUDT15 R139C* and TPMT^*^3C genotype distributions were in Hardy–Weinberg equilibrium (*p* = 0.50 and 0.85 respectively). Out of the 87 subjects, 59 subjects were *NUDT15 R139C* wild type (CC), which is nomal metabolizer (67.8%) and 27 subjects were *NUDT15 R139C* heterozygote (CT) (31.0%) while only one carried homozygote (TT) (1.1%) which is poor metabolizer. The mutant allelic T frequency of *NUDT15 R139C* was 16.7% (29/174). In the same cohort, 83 patients (95.4%) were wild type (TT) for TPMT^*^3C and 4 subjects (4.6%) were TPMT^*^3C heterozygote (TC), the allelic C frequency of TPMT^*^3C was 2.3% (Table 2).

**Table 2:** Allele distribution of NUDT15 R139C and TPMT^*^3C genotypes.

**Gene**	**Genotype**	**No**.	**Genotype frequency (%)**	**Allelic association**		
				**Allele**	**Allele frequency (%)**	**HWE *P*-value**
NUDT15 R139C	CC	59	67.8	C	83.3	0.50
	CT	27	31.0	T	16.7	
	TT	1	1.1			
TPMT*3C	TT	83	95.4	T	97.7	0.85
	TC	4	4.6	C	2.3	

*No., number of; HWE, Hardy–Weinberg equilibrium*.

### Association of *NUDT15 R139C* and TPMT^*^3C genotype with leukopenia

Of the 59 patients with wild type *NUDT15 R139C* (CC), only 5 (8.5%) suffered from leukopenia. Of the 27 patients with heterozygous *NUDT15 R139C* (CT), 17 patients (63.0%) developed leukopenia and one patient with the homozygotes (TT) (100%) suffered leukopenia and severe hair loss. The association of *NUDT15 R139C* with AZA-induced leukopenia was significant (*p* = 1.86 × 10^−7^). Compared with the wild type (CC), patients carrying variant allele T (CT + TT) had much higher risk in developing leukopenia (*p* = 1.79 × 10^−7^; OR = 7.59; 95%; CI, 3.16–18.21; Table 3). For the early and late phase of leukopenia, the influence of mutant allele T was significantly different, with OR and 95% CI as 8.85 (3.64–21.53), 3.93 (0.37–41.39), respectively (Table 4). *NUDT15 R139C* genotypes were significantly associated with early leukopenia (*p* = 1.25 × 10^−7^; Table 4), however no statistic association was observed for late leukopenia (*p* = 0.20). Among the 23 patients of leukopenia, 10 of them developed neutropenia. *NUDT15 R139C* was also strongly related with the incidence of neutropenia (43.5% mutation vs. 0% wild type, *p* = 3.78 × 10^−4^; OR = 12.21; 95% CI, 2.38–62.56). The patient with the *NUDT15 R139C* homozygote status (TT) and one patient with heterozygous (TC) developed severe hair loss, while no patients were observed with this phenomenon with the wild genotype (CC).

**Table 3:** Association of leukopenia with NUDT15 R139C and TPMT^*^3C genotypes.

**Gene**	**Genotype**	**Prevalence of leukopenia**	***p***	**Allelic association**			
				**Allele**	**Allele frequency**	***p***	**OR (95%Cl)**
NUDT15 R139C	CC	8.5%(5/59)	1.86 × 10^−7^	C	60.9%	1.79 × 10^−7^	7.59 (3.16–18.21)
	CT	63.0%(17/27)		T	39.1%		
	TT	100%(1/1)					
TPMT*3C	TT	26.5%(22/83)	0.95	T	97.8%	0.95	1.08 (0.11–10.65)
	TC	25%(1/4)		C	2.2%		

*OR, odds ratio; 95% CI, 95% confidence interval*.

**Table 4:** Association of different phase of leukopenia with NUDT15 R139C genotypes.

**Gene**	**Genotype frequency number (%)**				**Allelic association**		
**NUDT15 R139C**	**CC**	**CT**	**TT**	***p***	**Allele T frequency**	***p***	**OR (95%Cl)**
Leukopenia	5(8.5)	17(63.0)	1(100)	1.86 × 10^−7^	39.1%	1.79 × 10^−7^	7.59 (3.16–18.21)
Early leukopenia (< 8 w)	4(6.8)	16(59.3)	1(100)	1.25 × 10^−7^	42.9%	1.12 × 10^−7^	8.85 (3.64–21.53)
Late leukopenia (>8 w)	1(1.7)	1(3.7)	0	0.20	25%	0.22	3.93 (0.37–41.39)
controls	54(91.5)	10(37.0)	0		7.8%		

*OR, odds ratio; 95% CI, 95% confidence interval; controls, the patients without leukopenia; 8w, the eighth week*.

Of the 83 patients with wild type (TT) of TPMT^*^3C, 22 (26.5%) suffered leukopenia; of 4 patients with heterozygous (TC), 1 patient (25.0%) developed leukopenia. Thus, there was no significant association of TPMT^*^3C with AZA-induced leukopenia (*p* = 0.95; Table 3).

### ROC for an additive prediction model of leukopenia using *NUDT15 R139C*

**Figure 1:**
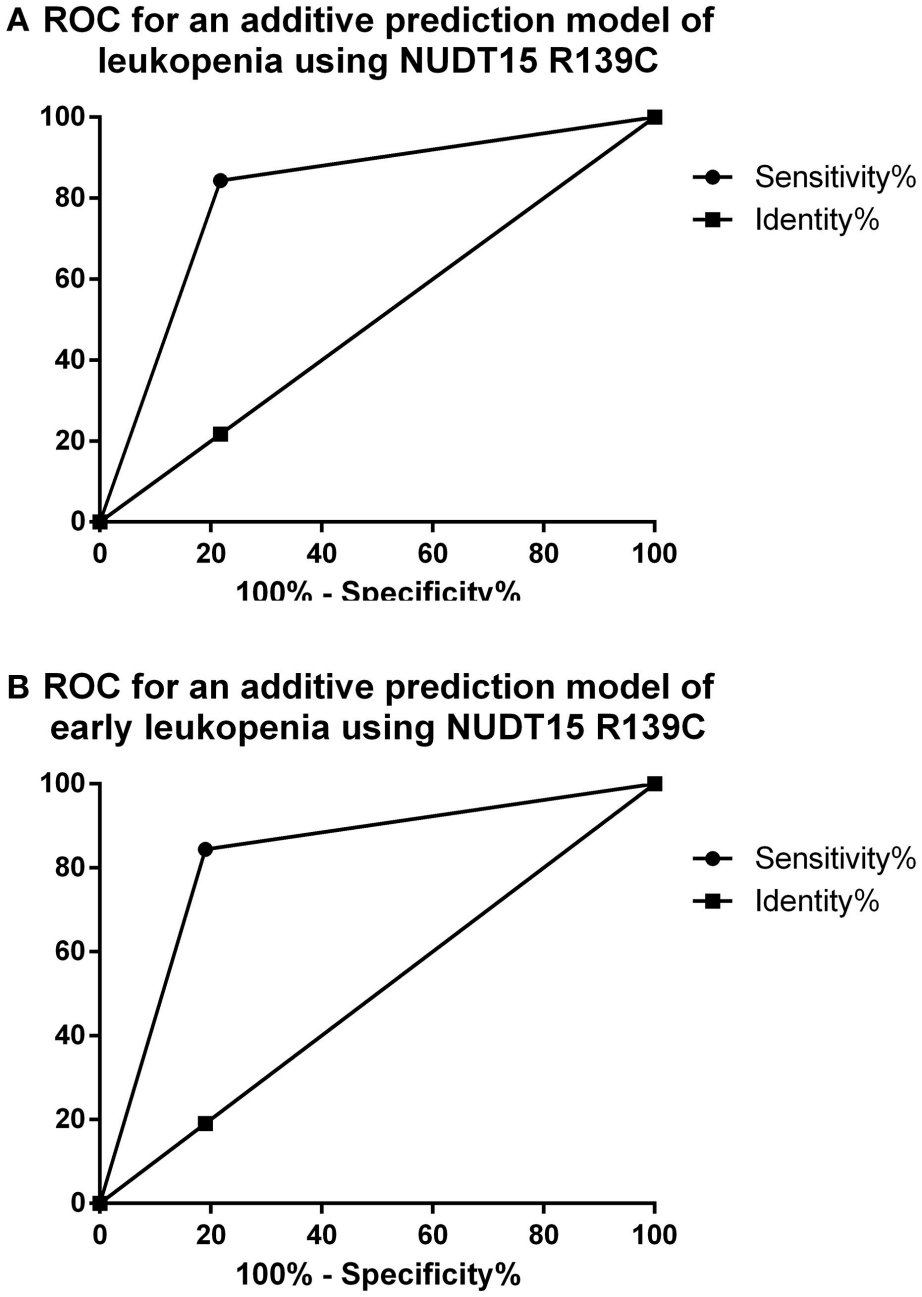
ROC for an additive prediction model of leukopenia and early leukopenia using NUDT15 R139C. **(A)** ROC for an additive prediction model of leukopenia using NUDT15 R139C. (Area under the ROC curve is 0.81, 95% confidence interval is 0.70–0.92 and *p*-value < 0.0001, the sensitivity and specificity were 84.4, 78.3% respectively). **(B)** ROC for an additive prediction model of early leukopenia using NUDT15 R139C. (Area under the ROC curve is 0.83, 95% confidence interval is 0.72–0.94 and *p*-value < 0.0001, the sensitivity and specificity were 84.4, 81.0% respectively).

We found that the *NUDT15 R139C* allele had a sensitivity of 84.4% and specificity of 78.3% for predicting leukopenia induced by AZA. Applying the ROC curve as an additive prediction model, the area under the curve with 95% CI 0.70–0.92 was 0.81. Using the *NUDT15 R139C* model to predict early leukopenia, the sensitivity and the specificity were 84.4, 81.0%, respectively, and the area under the curve with 95% CI 0.72–0.94 was 0.83 (Figure 1).

### Association of 6-TGN levels with leukopenia

The 6-TGN concentration of 87 patients ranged from 33.7 to 354.8 pmol/8^*^10^8^ RBC, mean (SD) 6-TGN concentration was 140.3 (62.38) pmol/8^*^10^8^ RBC. The associations of concentrations of 6-TGN with different variables were shown in Supplement Table S1. Overall, the level of 6-TGN was not significantly different between patients with leukopenia or the controls (*p* = 0.15), and no significant difference was observed between patients with early or late leukopenia (*p* = 0.12). Additionally, the 6-TGN concentration was not found to have a significant association with leukopenia in different genotypes, *NUDT15 R139C* and TPMT^*^3C genotypes (*p* = 0.62 and 0.25 respectively).

## Discussion

AZA is widely used as an immunosuppressive agent and its efficacy is endorsed by clinical studies. However, leukopenia, the most common ADR, still restricts its clinical application. In this study, we found a strong association of *NUDT15 R139C* with AZA-induced leukopenia, neutropenia and hair loss. It is an independent and first verification in Chinese autoimmune disease patients.

The incidence of leukopenia is about 5.0% based on the research on Caucasians (Ansari et al., 2008; Timmer et al., 2012), while the number is apparently higher in Asians. 35.4% (346/978) of Korean Crohn's disease (CD) patients at a median AZA dose of 1.7 mg/kg per day suffered leukopenia (Yang et al., 2014). 25.1% (34/135) of Japanese IBD patients had leukopenia at the AZA dose was 0.93 mg/kg per day (Kakuta et al., 2016) and in Chinese IBD patients, 18.1% (36/199) had observed leukopenia at a median AZA does of 50 mg/day (Gao et al., 2012). Higher frequency of severe leukopenia was observed in East-Asians, even if the AZA dose was lower than the recommended dosage.

The FDA recommend *TPMT* gene testing to prevent AZA-induced leukopenia before AZA treatment. *TPMT* plays a key role in the metabolism of AZA. Mutations cause abnormal metabolism and lead to accumulation of excess 6-TGN (Chouchana et al., 2014), which can increase the risk of AZA-induced adverse reactions, especially for leukopenia (Gearry et al., 2004). Paradoxically, AZA-induced leukopenia is more common in Asians, and compared with the 10.0% prevalence of *TPMT* reported in European population (Collie-Duguid et al., 1999). (Yang et al., 2014) demonstrated *TPMT* mutations in only 3.8% of Korean CD with leukopenia and (Zhu et al., 2016) reports only 4.6% Chinese individuals with IBD experiencing leukopenia carried variant *TPMT* alleles. There was only 4.6% of *TPMT* mutation prevalence among Chinese in our study which is similar to other reports in Asians (Ban et al., 2008; Lee et al., 2015). In our study, there were four patients (4.6%) who carried heterozygote mutant allele of TPMT^*^3C and only one patient (4.3%) with leukopenia carried *TPMT* variant alleles, however 22 patients (26.5%) with TPMT^*^3C wild type suffered leukopenia. Low mutant frequency of TPMT^*^3C in Asian population suggested this gene may have limited prediction value for AZA-induced leukopenia in Chinese patients. Although genetic polymorphism of *TPMT* has been recognized as a major risk factor for the development of AZA-induced myelotoxicity (Colombel et al., 2000), adverse events cannot be explained very well in Chinese patients by a normal or mutated *TPMT* genotype (Cao et al., 2009).

NUDT15 is a member of the nudix hydrolase enzyme family which mainly consists of pyrophosphohydrolases that act upon nucleoside diphosphates linked to other moieties, X (Bessman et al., 1996). (Moriyama et al., 2016) suggested that NUDT15 may prevent the incorporation of 6-thio-GTP (TGTP) and 6-thio-deoxyGTP (TdGTP) into DNA by dephosphorylating the thiopurine-active metabolites TGTP and TdGTP, thus negatively affecting the desired cytotoxic effects of AZA *in vivo* (Sandborn et al., 2000; Roberts and Barclay, 2015; Tanaka et al., 2015; Asada et al., 2016; Chiengthong et al., 2016; Kakuta et al., 2016; Liang et al., 2016; Moriyama et al., 2016; Park et al., 2016; Sato et al., 2016). *In-vitro* studies showed a higher percentage of apoptosis and necrosis in cells transfected with the *NUDT15 R139C* construction compared with cells with the wild type (Yang et al., 2014). In 2014, (Yang et al., 2014) showed there was a significant association between *NUDT15 R139C* and AZA-induced leukopenia in Korean IBD patients (*p* = 5.58 × 10^−43^, OR = 8.61). That was also verified in Japanese IBD patients, as the *NUDT15 R139C* was correlated with early leukopenia (*p* = 1.92 × 10^−16^, OR = 28.4; Kakuta et al., 2016). Our result is consistent with previous studies that *NUDT15 R139C* was associated with early leukopenia. However, in X. Zhu's research, *NUDT15 R139C* was not only associated with early leukopenia (0–8weeks) (*p* = 2.13 × 10^−19^, OR = 15.67) but also with middle (8–24weeks) and late (>24weeks) leukopenia (*p* = 4.51 × 10^−9^, OR = 12.06; *p* = 0.022, OR = 3.91 respectively) in Chinese IBD patients (Zhu et al., 2016). We enrolled 87 patients and the follow-up visiting was just 12 weeks which may limit our clinical results. Further studies using larger samples and longer follow-up visiting are needed to replicate our findings. Additionally, we also found that *NUDT15 R139C* was correlated with neutropenia (*p* = 3.78 × 10^−4^; OR = 12.21). (Kim et al., 2017) reported that the sensitivity was 85.7% and specificity was 92.2% by an additive prediction model of early leukopenia using *NUDT15 R139C* in Korean neurological diseases In our study, we calculated the predictability of *NUDT15* variant allele for early leukopenia and leukopenia respectively. The result showed that *NUDT15 R139C* variants predicted early leukopenia better (the sensitivity and the specificity were 84.4, 81.0%, respectively).

In addition, such association has been replicated in multiple independent follow-up studies and also demonstrated ethnic diversity. The *NUDT15 R139C* mutant allele frequency is high in Asians and Hispanics (about 10% in Asians), low in Caucasians (about 0.2%) and not found in Africans (Yang et al., 2015). *NUDT15 R139C* mutant frequency were 10.4 and 12% in Korean and Japanese IBD patients, respectively. In this study, we found that the frequency in Chinese autoimmune disease patients was about 32.1% which was higher than previous reports. The percentage of females was much higher than males (90.8 vs. 9.2%) in our study since females are more susceptible to autoimmune diseases like SLE, SS. We inferred the mutant frequency of *NUDT15 R139C* may be higher in female patients than males, but this hypothesis needs to be validated in further large sample studies. These studies, together with our data, suggest *NUDT15 R139C* may have a greater prediction ability than TPMT^*^3C genotyping for prospective risk assessment of AZA-induced leukopenia in Asian populations.

It is well-known that 6-TGN are the predominant active metabolites of AZA, and the accumulation of 6-TGN can induce adverse reactions, particularly for leukopenia (Armstrong et al., 2011). Monitoring 6-TGN blood concentrations routinely in patients receiving AZA has also been recommended (Dubinsky et al., 2000; Cuffari et al., 2004; Wright et al., 2004). The (Zhu et al., 2016) study suggested there was a slight difference in 6-TGN concentration between patients with or without leukopenia (*p* = 0.067), while 6-TGN levels were significantly correlated with leukopenia in the patients of *NUDT15* wild type (*p* = 0.0055). However, (Asada et al., 2016) reported there was no significant 6-TGN level difference in 161 Japanese IBD patients for *NUDT15* genotypes. In our research, we took the same measurement method of 6-TGN with previous studies in IBD, and no statistically significant concentration difference was observed between different *NUDT15* genotypes (*p* = 0.62). Among our 87 Chinese autoimmune disease patients, the 6-TGN levels ranged from 33.7 to 354.8 pmol/8^*^10^8^ RBC, mean (SD) was 140.3 (62.38) pmol/8^*^10^8^ RBC, which was significantly lower than the target 6-TGN monitoring range, 235-450 pmol/8^*^10^8^ RBC for IBD (Osterman et al., 2006). In patients without leukopenia, only three of them had 6-TGN levels within the recommend range (3/64), whose 6-TGN concentrations were 254.6, 277.2, 354.8 pmol/8^*^10^8^ RBC, respectively. There were also three (3/23) within the IBD range (240, 289.9, 347.5 pmol/8^*^10^8^ RBC, respectively) of patients with leukopenia. None of our patients were beyond the recommended 6-TGN monitoring range established for IBD. It indicates that the monitoring range for autoimmune disease patients needs to be established in the future. In addition (Moriyama et al., 2016), suggested that *NUDT15 R139C* may dephosphorylate the AZA-active metabolites TGTP and TdGTP rather than 6-TGN, which may explain why our concentrations were lower than the established range for IBD. The new therapeutic monitoring method detecting the metabolites TGTP and TdGTP may have more clinical value than 6-TGN.

In conclusion, we replicated previous findings that the *NUDT15 R139C* variant is a potential predictor for AZA-induced leukopenia in Chinese, extended this finding to patients with various autoimmune diseases and identified its specific association with leukopenia, especially for early leukopenia and neutropenia. Moreover, we found that the concentration of 6-TGN in autoimmune disease patients was much lower than the recommended range for IBD patients, suggesting a need for establishment of a new monitoring range for autoimmune disease patients. *NUDT15 R139C* gene testing may offer a more useful and effective way than *TPMT* to predict the AZA-induced leukopenia for physicians. They will promote the optimization of AZA medication in autoimmune disease patients.

## Ethics statement

This study was carried out in accordance with the recommendations of the ethical guidelines and approved by the research ethics committee of Drum Tower Hospital affiliated Nanjing University Medical School, Nanjing, China (2017-101-01) with written informed consent from all subjects. All subjects gave written informed consent in accordance with the Declaration of Helsinki. The protocol was approved by the ethical guidelines and approved by the research ethics committee of Drum Tower Hospital affiliated Nanjing University Medical School, Nanjing, China (2017-101-01).

## Author contributions

XF and QS: wrote the article; XF, QS, HZ, BH, SW, YF, and WG: designed the research; XF, HZ, BH, SW, and LG: performed the research; XF, and QS: analyzed the data.

### Conflict of interest statement

The authors declare that the research was conducted in the absence of any commercial or financial relationships that could be construed as a potential conflict of interest.
