# Supplementary material for: NUDT15 R139C Variants Increase the Risk of Azathioprine-Induced Leukopenia in Chinese Autoimmune Patients
Source: Front Pharmacol. 2018 May 7;9:460. doi: 10.3389/fphar.2018.00460 (PMC5949564; doi:10.3389/fphar.2018.00460)
Supplement: Supplementary file 1 [file Table_1.doc]

**Supplement Table S1 6-TGN concentration in different characteristics**

| Characteristics | 6-TGN  (pmol/8*10^8^RBC) | *p* |
| --- | --- | --- |
| Gender |  | 0.223 |
| female | 141.6 ± 64.65 |  |
| male | 127.7 ± 32.10 |  |
| AZA dose (mg/d) |  | 0.539 |
| 50 | 138.3 ± 63.99 |  |
| 100 | 155.7 ± 47.97 |  |
| leukopenia |  | 0.153 |
| With leukopenia | 140.8 ± 74.00 |  |
| Control | 140.1 ± 58.30 |  |
| Phrase of leukopenia |  | 0.119 |
| Eraly leukopenia | 140.7 ± 76.92 |  |
| Late leukopenia | 140.1 ± 46.32 |  |
| Control | 140.1 ± 58.30 |  |
| Disease |  | 0.625 |
| SLE | 131.6 ± 45.74 |  |
| SS | 159.8 ± 78.58 |  |
| Vasculitis | 129.8 ± 35.92 |  |
| Scleroderma | 135.5 ± 85.05 |  |
| DM | 148.3 ± 39.89 |  |
| CTD | 159.2 ± 135.26 |  |
| IgG4-RD | 96.0 ± 17.18 |  |
| AIH | 198.1 |  |
| NUDT15 |  | 0.624 |
| CC | 135.4 ± 60.30 |  |
| CT+TT | 150.6 ± 66.49 |  |
| TPMT*3C |  | 0.249 |
| TT | 141.4 ± 63.54 |  |
| TC | 118.6 ± 23.24 |  |

Abbreviations: AZA: azathioprine; Control: the patients without leukopenia; Early leukopenia: leukopenia happened within 8 weeks; Late leukopenia: leukopenia happened more than 8 weeks SLE: systemic lupus erythematosus; SS: Sjögren's syndrome; DM: dermatomyositis; CTD: connective tissue disease; IgG4-RD: IgG4 related diseases; AIH: autoimmune hepatitis
